# Integrative approaches to investigate the structure and assembly of *Trypanosoma brucei *BILBO1, a multidomain cytoskeletal protein at the flagellar pocket collar

**DOI:** 10.1186/2046-2530-4-S1-P25

**Published:** 2015-07-13

**Authors:** K Vidilaseris, B Morriswood, G Dong

**Affiliations:** 1 Department of Medical Biochemistry, Max F. Perutz Laboratories, Medical University of Vienna, Vienna, Austria; 2 Max F. Perutz Laboratories, University of Vienna, Vienna, Austria

## 

*Trypanosoma brucei *is a protist parasite and the causative agent of Human African Trypanosomiasis (sleeping sickness). At the base of its single flagellum is a bulb-like structure called the flagellar pocket (FP). The FP is the site of all endo-/exocytosis and thus essential for the survival of the parasite. At the neck of the FP is an electron-dense cytoskeletal structure termed the flagellar pocket collar (FPC), which currently has only one known protein component, BILBO1. Bioinformatic analysis indicates that there are four structural domains in the 67-kDa protein, including a globular N-terminal domain, two central EF-hand motifs followed by a long coiled-coil domain, and a C-terminal leucine zipper. *T. brucei *BILBO1 (TbBILBO1) by itself forms insoluble oligomers *in vitro*, which makes it intractable to any single conventional structural study method. We recently carried out structural dissection of TbBILBO1 using integrative structural biology approaches including NMR, crystallography, EM, and various biophysical methods. The high-resolution structure of its N-terminal domain reveals a variant ubiquitin-like fold with a conserved surface patch; mutagenesis of this patch causes cell death *in vivo*. We further found that the EF-hand motifs change their conformation upon calcium binding, the coiled-coil domain forms an antiparallel dimer, and intermolecular interactions between adjacent leucine zippers allow TbBILBO1 to form extended filaments *in vitro*. These filaments were additionally shown to condense into fibrous bundles through lateral interactions as demonstrated by our EM studies. Based on all these experimental data, we propose a mechanism for TbBILBO1 assembly into the flagellar pocket collar.

